# Food purchasing pattern is correlated to gender, educational level, and income: Results from the food purchasing habits survey

**DOI:** 10.1371/journal.pone.0322781

**Published:** 2025-06-05

**Authors:** Hiba F. Al-Sayyed, Zeinah F. AbuZeinah, Narmeen J. Al-Awwad

**Affiliations:** 1 Department of Nutrition, University of Petra, Amman, Jordan; 2 Department of Clinical Nutrition and Dietetics, The Hashemite University, Zarqa, Jordan; The World Islamic Sciences and Education University, JORDAN

## Abstract

Food purchasing pattern refers to the consumer behavior related to buying food. Rice and bread are the staple foods of Jordan. Legumes, cereals, milk, and dairy products constitute important part of the Jordanian diet. This cross-sectional study aimed to investigate food shopping patterns of people living in Jordan for milk, dairy products (e.g., yogurt, cheese), legumes and grains (rice, bulgur, *freekeh*, and couscous), and cereals (such as breakfast cereals, oatmeal, and granola). The study also aimed to understand the importance of certain consumer-related factors (like sociodemographic characteristics), product-specific factors (like taste, packaging, and origin (local *vs.* imported)), and external factors (like cost, brand name, freshness, and production/expiry dates) on the purchasing of these products. Most of the study participants were adult (18–30, n = 64.4%), educated (48.6%) females (75.6%). Female gender was correlated significantly (P < 0.05^*^) to purchasing local milk, dairy products and cereals due to freshness and famous brand names. In addition, socioeconomic status (indicated by household income and education level) affected significantly (P < 0.05) the type of milk and cereals purchased where price, freshness, participants’ perception of packaging and ingredient list, expiry date, and brand name affected purchasing of cereals and milk. Furthermore, age was found to be a significant (P < 0.05) determinant to buying local cereal products. Results of this investigation could be utilized by manufacturers and policy makers to improve the nutritional quality of food products offered in the Jordanian market, advertise for better local food choices, and to adopt focused strategies in order to develop the food sector in Jordan. Consumer behavior is a fundamental part of human daily lives. Studying consumer behavior pertains analyzing the factors affecting and affected buying goods.

## 1. Introduction

Jordan is one of the medium-low income, food-deficit [1] countries that have passed through several difficulties due to political conflicts in the surrounding areas. Jordan depends on imports [2] of 70% of staple foods [3]. In addition, the food processing sector including dairy and bakery manufacturing and packaging [4] contributes to Jordan’s food basket. Accordingly, the food supply in Jordan met consumer demands [5]. Nonetheless, food prices are increasing [6] necessitating the adoption of policies to stabilize and reduce food prices [7].

Food not only alleviates hunger but also, provides essential nutrition which determines humans health [8]. Food purchasing pattern is the major and sometimes the only driver for food acquisition in consumer-driven societies [9] and the key to extrapolating policies to improve human health, alleviate hunger, and improve the food system.

Consumer behavior refers to the practices related to buying in terms of the locations of purchasing, determinants of buying, and preferences [10,11]. Food purchasing pattern refers to the consumer behavior related to buying food. Consumer behavior is an integral part of human daily life [12]. Consumer purchasing behavior towards certain types of foods has been studied previously in Jordan [4,13–16] and worldwide [17,18].

Policies and practices that enhance the production, marketing, and advertising of affordable nutritious foods were always called from stakeholders and policymakers [19]. Consumer behavior and purchasing driver research are key to formulating such policies and practices [20].

Rice and bread are staple foods in Jordan. Legumes, other cereals, milk, and dairy products constitute an integral part of the Jordan food basket [21] coming in the second rank after animal-based products [4]. As a country that primarily relies on imports to meet its food demands [2], knowing the limitations of previously published reports in terms of modernity, method of data collection, and relevance to consumer’s dietary habits, nutritional and health status [11], and knowing the emphasized role of studying consumer behavior in shaping policies related to food systems, we sought to investigate the food shopping patterns of people living in Jordan for milk, dairy products (e.g., yogurt, cheese), legumes and grains (rice, bulgur, *freekeh*, and couscous), and cereals (such as breakfast cereals, oatmeal, and granola). The study also aimed to understand of the importance of certain consumer-related factors (like sociodemographic characteristics), product-specific factors (like taste, packaging, and origin (local *vs.* imported)), and external factors (like cost, brand name, freshness, and production/expiry dates) on the purchasing of these products in the year 2019.

### 1.1 Hypotheses

#### The null hypothesis of the research (H_0_).

There is no association between certain consumer-related factors (like sociodemographic characteristics), product-specific factors (like taste, packaging, and origin (local *vs.* imported)), and external factors (like cost, brand name, freshness, and production/expiry dates) on the purchasing of milk, dairy products (e.g., yogurt, cheese), legumes and grains (rice, bulgur, *freekeh*, and couscous), and cereals (such as breakfast cereals, oatmeal, and granola).

#### The alternative hypothesis (H_1_).

There is an association between certain consumer-related factors (like sociodemographic characteristics), product-specific factors (like taste, packaging, and origin (local *vs.* imported)), and external factors (like cost, brand name, freshness, and production/expiry dates) on the purchasing of milk, dairy products (e.g., yogurt, cheese), legumes and grains (rice, bulgur, *freekeh*, and couscous), and cereals (such as breakfast cereals, oatmeal, and granola).

## 2. Materials and methods

This study followed the Strengthening the reporting of observational studies in epidemiology (STROBE) guidelines [22] and Declaration of Helsinki of the World Medical Association [23].

### 2.1. Study design

This is an observational study of cross-sectional design.

### 2.2. Survey tool

Food purchasing pattern was assessed *via* a valid (with content coefficient of validity of 1.0), reproducible (Spearman-Brown factor correlation coefficient > 0.7) questionnaire developed previously by the authors. The questionnaire examines grocery shopping, meal preparation behaviors, and food label use affecting purchasing 13 food groups namely: bread, milk, other dairy products, rice, legumes, and other grains, cereals, snack foods, sweets, juices, and canned beverages, sugar, honey, and spreads, meat, poultry, eggs, and sea foods, fresh fruits and vegetables, canned and frozen fruits and vegetables. In addition, the questionnaire was developed in both Arabic and English language speakers [24].

### 2.3. Settings

This study was performed to include a representative sample of adult people living in Jordan who did grocery shopping for the household in the year 2019. The participants were asked for their demographic characteristics (sex, age, governorate of residence, marital status, household size, and income).

The exposure of interest was the purchasing pattern in the sense of the persons who are responsible for food preparation and shopping, location of shopping, time of shopping, usual shopping frequency and duration, and following a special diet. In addition, shopping characteristics of milk, dairy products (e.g., yogurt, cheese), legumes and grains (rice, bulgur, *freekeh*, and couscous), and cereals (such as breakfast cereals, oatmeal, and granola) were measured. Furthermore, the importance of consumer-related factors (like sociodemographic characteristics), product-specific factors (like taste, packaging, and origin (local *vs.* imported)), and external factors (like cost, brand name, freshness, and production/expiry dates) during purchasing of these products were also measured.

The survey tool was uploaded onto Google^®^ forms and a link was distributed online through email list serves and social media such as WhatsApp^®^ and Facebook^®^ between 18^th^ March 2019 and 30^th^ September 2019. Students at the Department of Nutrition/Faculty of Pharmacy and Medical Sciences/University of Petra/Amman/Jordan assisted with the distribution of the questionnaire.

### 2.4. Participants

The study population was selected conveniently from adults (older than 18 years) who live in Jordan and do grocery shopping for their households. Sample size calculation was performed online to be 384 [25] assuming a 95% confidence interval, alpha level of 0.05, and Jordanian population size of 10,832,645 [26].

### 2.5. Variables

The measured variables were: demographic characteristics (sex, age, governorate of residence, marital status, household size, and income), the purchasing pattern (the person who is responsible for food preparation, the person who is responsible for shopping, the place of shopping, time of shopping, usual shopping pattern (in terms of the shopping trip frequency and duration), and following a special diet. In addition, shopping characteristics of milk, dairy products (e.g., yogurt, cheese), legumes and grains (rice, bulgur, *freekeh*, and couscous), and cereals (such as breakfast cereals, oatmeal, and granola) were measured. Furthermore, the importance of consumer-related factors (like sociodemographic characteristics), product-specific factors (like taste, packaging, and origin (local *vs.* imported)), and external factors (like cost, brand name, freshness, and production/expiry dates) on the purchasing of milk, dairy products (e.g., yogurt, cheese), legumes and grains (rice, bulgur, *freekeh*, and couscous), and cereals (such as breakfast cereals, oatmeal, and granola). The measured variables were of qualitative type.

### 2.6. Data sources/measurement

To reduce respondent burden, questions were formulated to allow choosing the answer either dichotomous (sex, time of shopping,) or multiple-choice manner (age, governorate of residence, marital status, education, household size and income, shopping and meal pattern characteristics).

### 2.7. Bias

To limit bias, volunteers were asked to be accurate as much as possible. They were informed that participation in the study was voluntary, their withdrawal from answering the survey is free at any time. In addition, the data will be confidential, used in a blind manner (coded), and will be used only for scientific purposes.

### 2.8. Study size

Sample size calculation was performed online to be 384 [25] assuming 95% confidence interval, alpha level of 0.05, and Jordanian population size of 10,832,645 [26].

### 2.9. Statistical methods

Data were analyzed by the software: statistical package for social sciences (SPSS)^®^ 26^th^ version. Missing data were coded as (.). The variables were coded with Arabic figures. Rates and percentages for each variable were calculated as counts and ratios. In addition, the frequency and percentages of factors affecting shopping (i,e. consumer-related factors (like sociodemographic characteristics), product-specific factors (like taste, packaging, and origin (local *vs.* imported)), and external factors (like cost, brand name, freshness, and production/expiry dates)) within each food group (milk, dairy products (e.g., yogurt, cheese), legumes and grains (rice, bulgur, *freekeh*, and couscous), and cereals (such as breakfast cereals, oatmeal, and granola) were calculated. Chi-square tests were run and Pearson’s correlation coefficients were used to calculate the linear correlation between the variables. Significant (^*^) and highly significant (^**^) correlations were correlated when P-value < 0.05 and < 0.01 respectively.

### 2.10. Ethical approval

The survey tool was revised and approved by the Research Ethics Committee of the Faculty of Pharmacy and Medical Sciences, University of Petra, Amman, Jordan at the University of Petra (Decision number: (1H/1/2019). Participants were consented electronically by pressing the button (Yes) after reading a question in the survey (Do you agree to participate in this survey?). If they press (Yes), the survey begins. If they pressed (No), the potential participant goes out of the survey form.

## 3. Results

### 3.1. Demographic characteristics of the study participants

Table 1 shows the demographic characteristics of the study participants. The study consisted of 483 participants (~3:1 female: male ratio). The response rate for different questions ranged between 32.1% to 98.6% for males and females respectively. Most (75.1%) of the study participants were residents of Amman, married (54.1%), earned their bachelor’s degree (64.4%), aged between 18–30 (48.9%), with a household size larger than 5 individuals (34.8%), and 44.9% of the study participants had household income of less than 1000 JOD (1 JOD ~ 0.71 USD).

**Table 1:** Demographic characteristics of the study participants.

Characteristic	Number (Percentage)
Gender	
Female	365 (75.6)
Male	118 (24.4)
Age	
18-30	236 (48.9)
31-40	90 (18.6)
41-50	71 (14.7)
51-60	44 (9.1)
> 61	12 (2.5)
Unspecified	30 (6.2)
Governorate of residence	
Amman	364 (75.1)
Irbid	8 (1.7)
Zarqa	68 (16.1)
Madaba	2 (0.4)
Salt	10 (2.1)
Tafila	1 (0.2)
Aqaba	2 (0.4)
Karak	2 (0.4)
Mafraq	9 (1.9)
Jerash	5 (1.0)
Maa’an	1 (0.2)
Marital status	
Single	198 (41)
Married	264 (54.7)
Other	16 (3.3)
Not specified	5 (1)
Education	
Below secondary school	9 (1.9)
Secondary school	57 (11.8)
Bachelor	311 (64.4)
Diploma	63 (13)
Master	22 (4.6)
Philosophy doctorate/medical doctorate	17 (3.5)
Unspecified	1 (0.2)
Household size (persons)	
1	26 (5.4)
2	46 (9.5)
3	61 (12.6)
4	76 (15.7)
5	106 (21.9)
> 5	168 (34.8)
Household income (JOD)*	
< 500	82 (17)
500- < 1000	217 (44.9)
1000 – < 2000	106 (21.9)
2000 – < 3000	32 (6.6)
≥ 3000	29 (6)
Unspecified	17 (3.5)

### 3.2. Shopping and meal preparation characteristics of the study participants

Table 2 shows some shopping and meal-preparation characteristics of the study participants. Most (49.3%) of the study respondents prepared meals and did (37.9%) grocery shopping for their households from hypermarkets (71.9%) during the weekdays (61.3%) as a large trip once a week with a few small shopping trips during the week. In addition, most of the study participants did not follow a special dietary regimen (64.3%).

**Table 2:** Shopping and meal-preparation characteristics of the study participants.

Characteristic	Number (Percentage)
The person who is responsible for food preparation	
The respondent	238 (49.3)
Spouse	59 (12.2)
Nany or household help	3 (0.6)
Mother	159 (32.9)
Father	1 (0.2)
Food delivery or take out	8 (1.7)
Other	15 (3.1)
The person who is responsible for shopping	
The respondent	245 (50.7)
Spouse	70 (14.5)
Nany or household help	3 (0.6)
Mother	46 (9.5)
Father	90 (18.6)
Other	29 (6)
The place of shopping	
Hypermarkets	346 (71.6)
Small supermarkets	123 (25.5)
Online	2 (0.4)
Others	12 (2.5)
Time of shopping	
Weekdays	296 (61.3)
Weekends	187 (38.7)
Usual shopping pattern	
A large trip once a week with a few small shopping trips during the week	183 (37.9)
A large trip once every two weeks with a few small shopping trips	71 (14.7)
No big trips with small shopping as needed	90 (18.6)
A big weekly trip without small shopping	18 (3.7)
A big trip once a month with small shopping as needed	105 (21.7)
A large trip once every two weeks without small shopping	8 (1.7)
Other	8 (1.7)
Following a special diet (whether the respondent or any of the household members)	
Yes	168 (34.8)
No	313 (64.6)
Unspecified	2 (0.4)

Table 3 shows the shopping characteristics for milk, dairy products, rice, legumes, and other grains, in addition to the cereals food group such as breakfast cereals, oatmeal, and granola groups. Results show that most of the study participants purchase whole (full cream) milk (48.4%) and dairy products (71.6%). Furthermore, the study participants look at the expiry date of milk, yogurt, cheese, and dairy products, rice, legumes, bulgur, *freekeh*, couscous and other grains groups.

**Table 3:** Shopping characteristics related to different food groups.

Group	Characteristics	Number (Percentage)
Milk	Type of milk purchased	
	Skim	55 (11.4)
	Low	106 (21.9)
	Whole	234 (48.4)
	Vegan	0 (0)
	Other	56 (11.6)
	Don’t buy milk at all	32 (6.6)
	Looking at the expiry date	
	Never	20 (4.1)
	Rarely	13 (2.7)
	Sometimes	48 (9.9)
	Often	60 (12.4)
	Always	333 (68.9)
	Don’t buy milk at all	9 (1.9)
	Factors affecting purchasing	
	Taste	10 (2.1)
	Not important at all	12 (2.5)
	Not important	79 (16.4)
	Neutral	154 (31.9)
	Important	159 (32.9)
	Very important	69 (14.3)
	Unspecified	
	Packaging	64 (13.3)
	Not important at all	145 (30)
	Not important	127 (26.3)
	Neutral	58 (12)
	Important	20 (4.1)
	Very important	69 (14.3)
	Unspecified	
	Local	63 (13)
	Not important at all	139 (28.8)
	Not important	126 (26.1)
	Neutral	63 (13)
	Important	23 (4.8)
	Very important	69 (14.3)
	Unspecified	
	Imported	51 (10.6)
	Not important at all	130 (26.9)
	Not important	141 (29.2)
	Neutral	64 (13.3)
	Important	28 (5.8)
	Very important	69 (14.3)
	Unspecified	
	Cost	16 (3.3)
	Not important at all	66 (13.7)
	Not important	139 (28.8)
	Neutral	122 (25.3)
	Important	71 (14.7)
	Very important	69 (14.3)
	Unspecified	
	Freshness	13 (2.7)
	Not important at all	29 (6)
	Not important	95 (19.7)
	Neutral	129 (29.7)
	Important	80 (16.6)
	Very important	69 (14.3)
	Unspecified	
	Brand name	
	Not important at all	22 (4.6)
	Not important	56 (11.6)
	Neutral	111 (23)
	Important	143 (29.6)
	Very important	82 (17)
	Unspecified	69 (14.3)
	Production/expiry date	
	Not important at all	6 (1.2)
	Not important	4 (0.8)
	Neutral	33 (6.8)
	Important	71 (14.7)
	Very important	300 (62.1)
	Unspecified	69 (14.3)
Yogurt, cheese and other dairy products	Type of dairy product purchased	
	Skim	18 (3.7)
	Low	72 (14.9)
	Whole	346 (71.6)
	Unspecified	37 (7.7)
	Do not buy dairy products at all	10 (2.1)
	Looking at the expiry date	
	Never	13 (2.7)
	Rarely	14 (2.9)
	Sometimes	52 (10.8)
	Often	62 (12.8)
	Always	334 (69.2)
	Do not buy dairy products at all	8 (1.7)
	Factors affecting purchasing	
	Taste	7 (1.4)
	Not important at all	11 (2.3)
	Not important	81 (16.8)
	Neutral	154 (31.9)
	Important	183 (37.9)
	Very important	47 (9.7)
	Unspecified	
	Packaging	58 (12)
	Not important at all	142 (29.4)
	Not important	128 (26.5)
	Neutral	85 (17.6)
	Important	23 (4.8)
	Very important	47 (9.7)
	Unspecified	
	Local	45 (9.3)
	Not important at all	107 (22.2)
	Not important	148 (30.6)
	Neutral	95 (19.7)
	Important	41 (8.5)
	Very important	47 (9.7)
	Unspecified	
	Imported	45 (9.3)
	Not important at all	142 (29.4)
	Not important	156 (32.3)
	Neutral	63 (13)
	Important	30 (6.2)
	Very important	47 (9.7)
	Unspecified	
	Cost	19 (3.9)
	Not important at all	74 (15.3)
	Not important	149 (30.8)
	Neutral	137 (28.4)
	Important	57 (11.8)
	Very important	47 (9.7)
	Unspecified	
	Freshness	16 (3.3)
	Not important at all	25 (5.2)
	Not important	102 (21.1)
	Neutral	159 (32.9)
	Important	134 (27.7)
	Very important	47 (9.7)
	Unspecified	
	Brand name	25 (5.2)
	Not important at all	59 (12.2)
	Not important	129 (26.7)
	Neutral	135 (28)
	Important	88 (18.2)
	Very important	47 (9.7)
	Unspecified	
	Production/expiry date	6 (1.2)
	Not important at all	5 (1)
	Not important	41 (8.5)
	Neutral	75 (15.5)
	Important	309 (64)
	Very important	47 (9.7)
	Unspecified	
Rice, legumes, bulgur, *freekeh*, couscous and other grains	Buying	
	Yes	416 (86.1)
	No	26 (5.4)
	Unspecified	41 (8.5)
	Looking at the expiry date	
	Never	57 (11.8)
	Rarely	36 (7.5)
	Sometimes	69 (14.3)
	Often	77 (15.9)
	Always	232 (48)
	Do not buy these products at all	12 (2.5)
	Factors affecting purchasing	
	Taste	7 (1.4)
	Not important at all	19 (3.9)
	Not important	72 (14.9)
	Neutral	142 (29.4)
	Important	176 (36.4)
	Very important	67 (13.9)
	Unspecified	
	Packaging	46 (9.5)
	Not important at all	136 (28.2)
	Not important	133 (27.5)
	Neutral	73 (15.1)
	Important	28 (5.8)
	Very important	67 (13.9)
	Unspecified	
	Local	42 (8.7)
	Not important at all	127 (26.3)
	Not important	152 (31.5)
	Neutral	67 (13.9)
	Important	28 (5.8)
	Very important	67 (13.9)
	Unspecified	
	Imported	33 (6.8)
	Not important at all	126 (26.1)
	Not important	159 (32.9)
	Neutral	71 (14.7)
	Important	27 (5.6)
	Very important	67 (13.9)
	Unspecified	
	Cost	16 (3.3)
	Not important at all	61 (12.6)
	Not important	148 (30.6)
	Neutral	113 (23.4)
	Important	78 (16.1)
	Very important	67 (13.9)
	Unspecified	
	Freshness	17 (3.5)
	Not important at all	35 (7.2)
	Not important	120 (24.8)
	Neutral	144 (29.8)
	Important	100 (20.7)
	Very important	67 (13.9)
	Unspecified	
	Brand name	17 (3.5)
	Not important at all	64 (13.3)
	Not important	144 (29.8)
	Neutral	114 (23.6)
	Important	77 (15.9)
	Very important	67 (13.9)
	Unspecified	
	Production/expiry date	17 (3.5)
	Not important at all	64 (13.3)
	Not important	144 (29.8)
	Neutral	114 (23.6)
	Important	77 (15.9)
	Very important	67 (13.9)
	Unspecified	
Cereals such as breakfast cereals, oatmeal, granola, etc.	Buying	269 (55.7)
	Yes	177 (36.6)
	No	37 (7.7)
	Unspecified	
	Factors affecting purchasing	5 (1.0)
	Taste	7 (1.4)
	Not important at all	48 (9.9)
	Not important	100 (20.7)
	Neutral	109 (22.6)
	Important	214 (44.3)
	Very important	23 (4.8)
	Unspecified	
	Packaging	68 (14.1)
	Not important at all	97 (20.1)
	Not important	57 (11.8)
	Neutral	24 (5)
	Important	214 (44.3)
	Very important	34 (7)
	Unspecified	
	Local	83 (17.2)
	Not important at all	101 (20.9)
	Not important	38 (7.9)
	Neutral	13 (2.7)
	Important	214 (44.3)
	Very important	13 (2.7)
	Unspecified	
	Imported	66 (13.7)
	Not important at all	98 (20.3)
	Not important	55 (11.4)
	Neutral	37 (7.7)
	Important	214 (44.3)
	Unspecified	
	Cost	9 (1.9)
	Not important at all	35 (7.2)
	Not important	97 (20.1)
	Neutral	84 (17.4)
	Important	44 (9.1)
	Very important	214 (44.3)
	Unspecified	
	Freshness	7 (1.4)
	Not important at all	22 (4.6)
	Not important	73 (15.1)
	Neutral	87 (18)
	Important	80 (16.6)
	Very important	214 (44.3)
	Unspecified	
	Brand name	10 (2.1)
	Not important at all	25 (5.2)
	Not important	69 (14.3)
	Neutral	100 (20.7)
	Important	65 (13.5)
	Very important	214 (44.3)
	Unspecified	

Results of this study show that the factors that affected the above-mentioned food group purchasing were: taste, cost, freshness, brand name, and production/expiry dates. Tables 4–7 show the significantly correlated dependent (related to food purchasing pattern) and independent variables (gender, household income, educational level, and age), their distribution (as frequency), and P-value for the correlation. Female gender was correlated significantly (P < 0.05^*^) to purchasing local milk, dairy products, cereals, and cereals due to freshness and brand name. Moreover, gender is highly (P < 0.01^**^) correlated to the type of purchased milk (Table 4).

**Table 4:** The significant correlation coefficients between gender and the studied dependent variables related to food purchasing pattern.

Food group	Dependent variable	Female: male ratio (actual frequency)	P-value
Milk	Type purchased		0.000^**^
	Skim (non-fat)	2:1 (37:18)	
	Low (reduced fat)	7:1 (92:14)	
	Whole (full fat, regular)	3:1 (180:54)	
	Soy/almond milk	0:1 (0:1)	
	Other	1:1 (16:16)	
	Do not buy mil at all	2:1 (40:16)	
	Being local product		0.037^*^
	Not important at all	4:1 (51:12)	
	Not important	5:1 (117:22)	
	Neutral	3:1 (95:31)	
	Important	4:1 (49:14)	
	Very important	1:1 (13:10)	
Yogurt, cheese and other dairy products	Being local product		0.023^*^
	Not important at all	4:1 (36:9)	
	Not important	5:1 (90:17)	
	Neutral	2:1 (111:37)	
	Important	3:1 (73:22)	
	Very important	1:1 (24:17)	
Cereals such as breakfast cereals, oatmeal, granola, etc.	Being local product		0.028^*^
	Not important at all	2:1 (22:12)	
	Not important	7:1 (73:10)	
	Neutral	4:1 (79:22)	
	Important	4:1 (31:7)	
	Very important	1:1 (8:5)	
	Freshness		0.030^*^
	Not important at all	3:1 (5:2)	
	Not important	2:1 (15:7)	
	Neutral	4:1 (58:15)	
	Important	9:1 (78:9)	
	Very important	2:1 (57:23)	
	Brand name		0.044^*^
	Not important at all	1:1 (5:5)	
	Not important	24:1 (24:1)	
	Neutral	3:1 (55:14)	
	Important	3:1 (77:23)	
	Very important	4:1 (52:13)	

**Table 5:** The significant correlation coefficients between household income and the studied dependent variables related to food purchasing pattern.

Food group	Dependent variable	Frequency (percentage)	P-value
Milk	Type purchased		0.000**
	Skim (non-fat)		
	<500 JD		
	500- < 1000 JD	10 (18.5)	
	1000- < 2000 JD	17 (31.5)	
	2000- < 3000 JD	13 (24.1)	
	≥3000 JD	8 (14.8)	
	Low (reduced fat)	6 (0.1)	
	<500 JD		
	500- < 1000 JD	10 (10.2)	
	1000- < 2000 JD	47 (48)	
	2000- < 3000 JD	30 (30.6)	
	≥3000 JD	9 (9.2)	
	Whole (full fat, regular)	2 (2)	
	<500 JD		
	500- < 1000 JD	46 (0.4)	
	1000- < 2000 JD	115 (0.5)	
	2000- < 3000 JD	51 (22.2)	
	≥3000 JD	7 (3)	
	Other	12 (5.2)	
	<500 JD		
	500- < 1000 JD	12 (40)	
	1000- < 2000 JD	12 (40)	
	2000- < 3000 JD	3 (10)	
	≥3000 JD	2 (6.6)	
	Do not buy milk at all	1 (3.3)	
	<500 JD		
	500- < 1000 JD	5 (9.4)	
	1000- < 2000 JD	26 (49)	
	2000- < 3000 JD	9 (16.9)	
	≥3000 JD	6 (11.3)	
Cereals such as breakfast cereals, oatmeal, granola, etc.	Purchasing	8 (15)	0.002**
	Yes		
	<500 JD		
	500- < 1000 JD	36 (14)	
	1000- < 2000 JD	114 (5.4)	
	2000- < 3000 JD	64 (24.8)	
	≥3000 JD	23 (8.9)	
	No	21 (8.1)	
	<500 JD		
	500- < 1000 JD	43 (24.9)	
	1000- < 2000 JD	85 (49.1)	
	2000- < 3000 JD	35 (20.2)	
	≥3000 JD	7 (4)	
	The packaging looks nice/ The product comes bundled with a gift	4 (2.3)	0.029**
	Not important at all		
	<500 JD		
	500- < 1000 JD	7 (33.3)	
	1000- < 2000 JD	7 (33.3)	
	2000- < 3000 JD	5 (23.8)	
	≥3000 JD	1 (4.8)	
	Not important	1 (4.8)	
	<500 JD		
	500- < 1000 JD	6 (9)	
	1000- < 2000 JD	29 (43.3)	
	2000- < 3000 JD	15 (22.4)	
	≥3000 JD	7 (10.4)	
	Neutral	10 (15)	
	<500 JD		
	500- < 1000 JD	10 (10.8)	
	1000- < 2000 JD	45 (48.4)	
	2000- < 3000 JD	29 (31.2)	
	≥3000 JD	5 (5.4)	
	Important	4 (4.3)	
	<500 JD		
	500- < 1000 JD	6 (11.3)	
	1000-< 2000 JD	25 (47.2)	
	2000- < 3000 JD	13 (24.5)	
	≥3000 JD	6 (11.3)	
	Very important	3 (5.7)	
	<500 JD		
	500- < 1000 JD	7 (29.2)	
	1000- < 2000 JD	8 (33.3)	
	2000- < 3000 JD	2 (8.3)	
	≥3000 JD	4 (16.7)	
		3 (12.5)	

**Table 6:** The significant correlation coefficients between educational level and the studied dependent variables related to food purchasing pattern.

Food group	Dependent variable	Frequency (percentage)	P-value
Milk cost	Cost as a factor that determines purchasing		0.047*
	Not important at all	0 (0)	
	High school	5 (31.2)	
	Diploma (2-years post-secondary school)	8 (50)	
	Bachelor	3 (18.8)	
	More than bachelor		
	Not important		
	High school	4 (6.2)	
	Diploma (2-years post-secondary school)	5 (7.8)	
	Bachelor	46 (71.9)	
	More than bachelor	9 (13.4)	
	Neutral		
	High school	16 (11.6)	
	Diploma (2-years post-secondary school)	16 (11.6)	
	Bachelor	93 (67.4)	
	More than bachelor	13 (9.4)	
	Important		
	High school	13 (10.8)	
	Diploma (2-years post-secondary school)	13 (10.8)	
	Bachelor	83 (69.2)	
	More than bachelor	11 (9.2)	
	Very important		
	High school	12 (17.4)	
	Diploma (2-years post-secondary school)	11 (15.9)	
	Bachelor	45 (26.6)	
	More than bachelor	1 (0.6)	
Milk brand name	Brand name as a factor determine purchasing		0.019**
	Not important at all	1 (4.5)	
	High school	6 (27.3)	
	Diploma (2-years post-secondary school)	11 (50)	
	Bachelor	4 (18.2)	
	More than bachelor		
	Not important		
	High school	5 (9.4)	
	Diploma (2-years post-secondary school)	7 (13.2)	
	Bachelor	36 (67.9)	
	More than bachelor	5 (9.4)	
	Neutral		
	High school	9 (8.2)	
	Diploma (2-years post-secondary school)	7 (6.4)	
	Bachelor	87 (79.1)	
	More than bachelor	7 (6.4)	
	Important		
	High school	21 (14.9)	
	Diploma (2-years post-secondary school)	20 (14.2)	
	Bachelor	82 (58.2)	
	More than bachelor	18 (12.8)	
	Very important		
	High school	9 (11.1)	
	Diploma (2-years post-secondary school)	10 (12.3)	
	Bachelor	59 (72.8)	
	More than bachelor	3 (3.7)	
Milk type	Type purchased		0.029*
	Skim (non-fat)		
	High school	11 (20)	
	Diploma (2-years post-secondary school)	1 (1.8)	
	Bachelor	35 (63.7)	
	More than bachelor	8 (14.5)	
	Low (reduced fat)		
	High school	10 (9.8)	
	Diploma (2-years post-secondary school)	12 (11.7)	
	Bachelor	70 (68.6)	
	More than bachelor	10 (9.8)	
	Whole (full fat, regular)		
	High school	24 (10.4)	
	Diploma (2-years post-secondary school)	32 (1.3)	
	Bachelor	158 (68.4)	
	More than bachelor	17 (7.4)	
	Other		
	High school	3 (9.7)	
	Diploma (2-years post-secondary school)	5 (16.1)	
	Bachelor	21 (67.7)	
	More than bachelor	2 (6.5)	
	Do not buy milk at all		
	High school	9 (17.6)	
	Diploma (2-years post-secondary school)	13 (25.5)	
	Bachelor	27 (52.9)	
	More than bachelor	2 (3.9)	
	Brand name as a factor determine purchasing		0.019*
	Not important at all		
	High school	1 (4.5)	
	Diploma (2-years post-secondary school)	6 (27)	
	Bachelor	11 (50)	
	More than bachelor	4 (18.2)	
	Not important		
	High school	5 (9.4)	
	Diploma (2-years post-secondary school)	7 (13.2)	
	Bachelor	36 (67.9)	
	More than bachelor	5 (9.4)	
	Neutral		
	High school	9 (8.1)	
	Diploma (2-years post-secondary school)	7 (6.4)	
	Bachelor	87 (79.1)	
	More than bachelor	7 (6.4)	
	Important		
	High school	21 (14.9)	
	Diploma (2-years post-secondary school)	20 (14.2)	
	Bachelor	82 (58.2)	
	More than bachelor	18 (12.8)	
	Very important		
	High school	9 (11.1)	
	Diploma (2-years post-secondary school)	10 (12.3)	
	Bachelor	59 (72.8)	
	More than bachelor	3 (3.7)	
Yogurt, cheese and other dairy products	Taste as a factor determine purchasing		0.001**
	Not important at all		
	High school	0 (0)	
	Diploma (2-years post-secondary school)	2 (28.6)	
	Bachelor	2 (28.6)	
	More than bachelor	3 (42.9)	
	Not important		
	High school	4 (36.3)	
	Diploma (2-years post-secondary school)	3 (27.3)	
	Bachelor	4 (36.3)	
	More than bachelor	0 (0)	
	Neutral		
	High school	11 (13.9)	
	Diploma (2-years post-secondary school)	10 (12.7)	
	Bachelor	54 (68.4)	
	More than bachelor	4 (5.1)	
	Important		
	High school	10 (6.5)	
	Diploma (2-years post-secondary school)	21 (13.6)	
	Bachelor	105 (68.6)	
	More than bachelor	18 (11.8)	
	Very important		
	High school	22 (12.4)	
	Diploma (2-years post-secondary school)	17 (9.6)	
	Bachelor	125 (70.6)	
	More than bachelor	13 (7.3)	
Expiry date	Production/expiry date as a factor determine purchasing		0.029*
	Not important at all		
	High school	0 (0)	
	Diploma (2-years post-secondary school)	2 (40)	
	Bachelor	1 (20)	
	More than bachelor	2 (40)	
	Not important		
	High school	2 (40)	
	Diploma (2-years post-secondary school)	0 (0)	
	Bachelor	2 (40)	
	More than bachelor	1 (20)	
	Neutral		
	High school	6 (15)	
	Diploma (2-years post-secondary school)	7 (17.5)	
	Bachelor	26 (65)	
	More than bachelor	1 (2.5)	
	Important		
	High school	4 (5.4)	
	Diploma (2-years post-secondary school)	10 (13.5)	
	Bachelor	52 (70.3)	
	More than bachelor	8 (10.8)	
	Very important		
	High school	35 (11.5)	
	Diploma (2-years post-secondary school)	34 (11.2)	
	Bachelor	209 (68.8)	
	More than bachelor	26 (8.6)	

**Table 7:** The significant correlation coefficients between age and the studied dependent variables related to food purchasing pattern.

Food group	Dependent variable	Frequency (percentage)	P-value
Milk	Being local product		0.003**
	18-30		
	Not important at all	7 (63.7)	
	Not important	2 (18.2)	
	Neutral	1 (9.1)	
	Important	1 (9.1)	
	Very important	0 (0)	
	31-40		
	Not important at all	16 (57.1)	
	Not important	7 (25)	
	Neutral	3 (10.7)	
	Important	2 (7.1)	
	Very important	0 (0)	
	41-50		
	Not important at all	46 (50)	
	Not important	23 (25)	
	Neutral	14 (15.2)	
	Important	7 (7.6)	
	Very important	2 (2.2)	
	51-60		
	Not important at all	63 (53.3)	
	Not important	28 (23.7)	
	Neutral	17 (14.4)	
	Important	9 (7.6)	
	Very important	1 (0.8)	
	61+		
	Not important at all	68 (48.9)	
	Not important	23 (16.5)	
	Neutral	26 (18.7)	
	Important	16 (11.5)	
	Very important	6 (4.3)	
	Freshness		0.005**
	18-30		
	Not important at all	7 (63.6)	
	Not important	2 (18.2)	
	Neutral	1 (9.1)	
	Important	1 (9.1)	
	Very important	0 (0)	
	31-40		
	Not important at all	16 (57.1)	
	Not important	7 (25)	
	Neutral	3 (10.7)	
	Important	2 (7.1)	
	Very important	0 (0)	
	41-50		
	Not important at all	46 (50)	
	Not important	23 (25)	
	Neutral	14 (15.2)	
	Important	7 (7.6)	
	Very important	2 (2.2)	
	51-60		
	Not important at all	63 (53.3)	
	Not important	28 (23.7)	
	Neutral	17 (14.4)	
	Important	9 (7.6)	
	Very important	1 (0.8)	
	61+		
	Not important at all	68 (48.9)	
	Not important	23 (16.5)	
	Neutral	26 (18.7)	
	Important	16 (11.5)	
	Very important	6 (4.3)	
Yogurt, cheese and other dairy products	Being local		0.007**
	18-30		
	Not important at all	26 (63.4)	
	Not important	7 (17.1)	
	Neutral	5 (12.2)	
	Important	3 (7.3)	
	Very important	0 (0)	
	31-40		
	Not important at all	54 (53.5)	
	Not important	21 (20.8)	
	Neutral	15 (14.9)	
	Important	10 (9.9)	
	Very important	1 (0.1)	
	41-50		
	Not important at all	78 (55.3)	
	Not important	32 (22.7)	
	Neutral	23 (16.3)	
	Important	7 (5)	
	Very important	1 (0.7)	
	51-60		
	Not important at all	36 (40.9)	
	Not important	19 (21.6)	
	Neutral	14 (15.9)	
	Important	12 (13.6)	
	Very important	7 (8)	
	61+		
	Not important at all	19 (51.4)	
	Not important	6 (16.2)	
	Neutral	6 (16.2)	
	Important	5 (13.5)	
	Very important	1 (2.7)	
Cereals such as breakfast cereals, oatmeal, granola, etc.	Being local		0.043**
	18-30		
	Not important at all	15 (55.5)	
	Not important	5 (18.5)	
	Neutral	5 (18.5)	
	Important	2 (7.4)	
	Very important	0 (0)	
	31-40		
	Not important at all	37 (47.4)	
	Not important	22 (28.2)	
	Neutral	15 (19.2)	
	Important	4 (5.1)	
	Very important	0 (0)	
	41-50		
	Not important at all	52 (55.9)	
	Not important	22 (23.7)	
	Neutral	13 (14)	
	Important	4 (4.3)	
	Very important	2 (2.2)	
	51-60		
	Not important at all	14 (36.8)	
	Not important	7 (18.4)	
	Neutral	10 (26.3)	
	Important	4 (10.5)	
	Very important	3 (7.9)	
	61+		
	Not important at all	7 (53.8)	
	Not important	2 (15.4)	
	Neutral	0 (0)	
	Important	3 (23.1)	
	Very important	1 (7.7)	

Table 5 shows a highly significant (P < 0.01^**^) correlation between the household income level and the type of bought milk, buying cereals such as breakfast cereals, oatmeal, granola, and participants’ perception about packaging as a factor affecting their purchasing of cereals such as breakfast cereals, oatmeal, granola.

Table 6 shows that there is a significant (P < 0.05^*^) correlation between educational level and the type of milk purchased and ingredient list as a factor affecting cereal purchasing. In addition, there is a highly significant (P < 0.01^**^) correlation between education level and dairy expiry date and taste as well as milk brand name and cost as factors affecting milk purchasing. Furthermore, education level affected the type of milk purchased significantly (P < 0.05^*^).

Table 7 shows that there is a significant (P < 0.05^*^) correlation between age and buying local cereal products. In addition, there is a highly significant (P < 0.01^**^) correlation between age and purchasing local milk, yogurt, cheese, and other dairy products, as well as freshness as a factor contributing to buying milk.

## 4. Discussion

In this investigation, we used (Jordanian Food Purchasing Habits Survey); a previously developed, validated (with content coefficient of validity of 1.0) questionnaire that was tested for reproducibility (Spearman-Brown factor correlation coefficient > 0.7), published and distributed in both Arabic and English languages [27]. Data collection *via* questionnaires distributed in Arabic and English allows the accommodation of different language cultures of sample.

### 4.1. Demographic and meal preparation characteristics of the study participants

The male: female ratio in Jordan is approximately 1:1. The high female: male ratio observed in the response to the survey probably indicates that females are more likely to do household grocery shopping [28] and to respond to questionnaire surveys. Worldwide, women respond more than males to surveys [29–31]. Additionally, in Jordan, women usually head the household [32].

Youth of age 18–23 years comprise about 1/3^rd^ of Jordanian population [33]. In 2020, the average monthly salary of Jordanians was reported to be 408 JOD/person [34]. The average household size in Jordan is 4.7 [35]. Jordan population is well-educated [4,36] with literacy rate of Jordanian youth is 98.3% [37]. The range of response rate of different questions is expected since answering the questionnaire was voluntary and some people were uncomfortable answering questions about confidential information like household income. In addition, most of Jordan population resides in Amman [38].

Since most of the study participants were females, they prepared meals and did grocery shopping for their households. Worldwide, women cook [39–41] and do grocery shopping [28,42–44] more than men. The finding that our study participants did not follow a special diet is consistent with the fact that Jordanians have the best health status among all of the Middle Eastern nations [45]. Thus, even though our sample was convenient, it represented the Jordanian population in terms of age, the governorate of residence, household size, and income.

### 4.2. Shopping characteristics of the study participants

Unlike Americans who shop for groceries several times *per* week [46] from non-traditional formats [47], our results (Table 1) show that the study sample shopped for groceries once *per* week from hypermarkets and did other several small trips during the week if needed. Bawa et al. [48] showed a negative correlation between shopping frequency and the *per* trip expenses. Thus, it seems that Jordanians attempted to save costs by shopping from hypermarkets since Jordan was classified as middle-income country [49]. In addition, shopping pattern shown by our results shopping pattern was cited by [9] as a tag for well-educated consumers; a characteristic found in our study sample characteristics (Table 1).

Consumer behavior is a complicated relationship that is affected by consumer-related factors such as biological needs, genetic profiles [7], and social and cultural factors [50]. In addition, food purchasing is strongly affected by product-specific factors and external factors [7].

Gender is one of the sociodemographic characteristics cited in the literature as a major factor affecting purchasing power, awareness [51], and healthier food preferences [7]. Within this context, we noticed that whole milk and dairy products were purchased mostly by females (Table 3). In addition, the female gender was correlated significantly to purchasing local milk, dairy products, cereals, and cereals due to freshness and brand name. Moreover, female gender was highly (P < 0.01^**^) correlated to the type of purchased milk (Table 4). Our results are somewhat similar to those of Fenech [28] where female gender, price, being domestic products, and brand name were identified as the major drivers for purchasing by females. In addition, Krešić [52] found that the female gender was significantly correlated to buying low-fat dairy products. These investigators also found that taste, sensory aspects, and health aspects were very important determinants of buying dairy products and this is consistent with our findings where 47.6% of people found taste to be an important or very important factor in their decision to purchase dairy products. Within the same context, Arganini [50,52] and Krešić [52] found that females tend to purchase foods especially dairy products according to health aspects. Low-fat products might be chosen more by females probably due to the fact that females concern more about their weight. In terms of cereals, Binkley [53] showed that females (especially if they were not employed) buy healthier breakfast cereals. Furthermore, US, UK, as well as Canadian populations were found to buy locally made groceries. The authors attributed this to flexible delivery, better response time during customer services and lower impact on the environment [54]. Within the same line, knowing the several physiological, psychological, and economic factors that affect consumer behavior, the random utility model showed that the commodity benefit (in terms of healthfulness) is one of the major drivers for purchasing [11]. Similarly, Tseng [9] emphasized that consumers with higher socio-economic status (as indicated by higher educational level) usually tend to buy food of low-fat and higher-fiber foods regardless of the price.

Our results (Table 5) showed that the higher the socioeconomic status of the study participants, the higher the purchasing of healthier milk and dairy product choices, breakfast cereals, oatmeal, granola, and the participant’s perception of packaging. This could be attributed to higher educational level and awareness about the importance of food packaging pertaining food label [11,55] as well as better food choices [9,56]. Higher education was cited in the literature as an indicator for higher socioeconomic status [9]. In Jordan, Rabboh et[4] reported that the higher-income households consume more processed foods with about 30–40% of the household income spent on animal-origin foods including dairy products. This can be confirmed by the knowledge, attitudes, and practices (KAP) model where knowledge affects the practices of purchasing [11].

Consistent with our results, Kurajdova [57] found that Slovakians buy milk according to income. Consistent with this, income was found to be a significant determinant of milk purchasing [11,57,58] and a major factor affecting food accessibility [59]. Within the same line, income was found to affect purchasing of breakfast cereals [60]. It is not surprising that income affected purchasing pattern since the higher the income, the higher the proportion used for grocery shopping [61]. On the other hand, Turrell [62] showed that reduced household income was associated with purchasing healthier foods. Moreover, income was shown to be a very strong predictive factor for food purchasing pattern especially for cereals, bread, and milk [63]. Packaging is known to affect consumer behavior by capturing attention of the consumers, motivating impulse buying, and providing attraction. In addition, labelling affects consumer behavior [64,65]. Since local products are being purchased significantly, Jordanian government, policy makers, and manufacturers are called to adopt local food firm advertising and marketing policies. Among the most important physical resources identified by the food choice model is money, where the household income determines social frameworks, roles, and meanings for the whole household [11].

We found a significant correlation between level of education, purchased milk type, and ingredient list as an important factor that affected cereal purchasing (Table 6). Concomitant with this, education was found to shape grocery purchasing behavior [51] *via* affecting lifestyle, satisfaction, and buying intentions [66]. Educated people tend to be more aware about the importance of reading the food label and ingredient list as well as production and expiration dates [67]. In addition, educated consumers are probably more worried about their health [56] and more able to afford purchasing more expensive food choices [9,55]

Our results indicate that age was correlated significantly with purchasing local milk and dairy products. Also, freshness is correlated to purchasing cereal products (Table 7). In general, age is considered a personal determinant of shaping [51] food purchasing specially milk [58,68,69] and fermented milk product [52] purchasing. Within the same line, consumer age was found by Mortimer and Clarke [42] as a determinant for purchasing in terms of the importance of product characteristics upon purchasing. Fortunately, Jordan has self-efficacy in fresh products [4]. In addition, age was documented as a factor that is related to appreciating the consumption of more fresh products [7].

## 5. Conclusions

It can be concluded that gender, household income, educational level, and age affected the purchasing of milk, yogurt, cheese, and dairy products, rice, legumes, bulgur, *freekeh*, couscous and other grains, legumes, and cereals such as breakfast cereals, oatmeal, granola products of Jordanians. These results imply accepting the alternative hypothesis and rejecting the null hypothesis.

Though consumer behavior is a complex uneasily understood relationship, it is somehow predictable and could be utilized by manufacturers and policy makers in order to improve the nutritional quality of food products offered in the Jordanian market, advertise for better local food choices, and to adopt focused strategies in order to develop the food sector in Jordan.

## 5. Limitations

Even though this is the first investigation of its kind, it is not without limitations such as convenience sampling procedure where there is a chance for sampling and the inability for generalization beyond the study sample. In addition, the cross-sectional design limits the generalization of the investigation results to the study population only.

## Supporting information

S1 Data XXX. (XLSX)
